# Incorporation of Coumarin-Based Fluorescent Monomers into Co-Oligomeric Molecules

**DOI:** 10.3390/polym10040396

**Published:** 2018-04-03

**Authors:** Edgar Teixeira, João C. Lima, A. Jorge Parola, Paula S. Branco

**Affiliations:** LAQV-REQUIMTE, Departamento de Química, Faculdade de Ciências e Tecnologia, Universidade NOVA de Lisboa, 2829-516 Caparica, Portugal; eh.teixeira@campus.fct.unl.pt (E.T.); lima@fct.unl.pt (J.C.L.)

**Keywords:** vinylcoumarins, fluorescent polymers, oligomers

## Abstract

With the purpose of modifying organic fluorescent dyes based on the coumarin scaffold, and developing and evaluating a route to its incorporation into a polymeric backbone, a study was conducted on the co-polymerization of 3-vinylcoumarins with styrene and methyl acrylate using 2,2-azobis(isobutyronitrile) (AIBN) as the radical initiator. The structural and photophysical characterization proved the incorporation of the coumarin monomers into the polymeric chain and further showed a decrease in the fluorescence quantum yields in the co-oligomers.

## 1. Introduction

The incorporation of fluorescent monomers into polymeric/oligomeric design molecules is an area of intense research nowadays due to their diverse potential applications in organic electronic devices (OLEDs) [1] as well as in sensor molecules for biochemical and environmental applications [2,3]. In one approach, the dye may be incorporated into polymer backbones as a sidechain by direct co-polymerization being the dye part of the core structure and not of the surface of the particle, resulting in an enhanced fluorescence of the particles [4,5]. For bio-imaging purposes, a large number of organic dyes such as fluorescein, rhodamine and coumarin dyes have been developed and incorporated into polymeric architecture to examine fundamental processes at tissue, cellular, and molecular level [6,7]. Several studies have shown that coumarins may present different behavior under acidic and basic conditions, which makes this a monomer with great potential. Applications involving coumarin based-polymers includes their use as fluorescent sensors for metal ion detection which have been very recently reported showing reasonable sensitivity and selectivity towards Cu(II) [8] or Hg(II) [9]. A wide range of other applications focuses on electronic devices [10,11,12] or on stimuli-responsive polymers such as photo-, temperature-, or pH-responsive [13,14]. Also the photoreactive coumarin ring anchored into homopolymers or co-polymer containing acrylic and methacrylic moieties allowed the formation of thermo- and photo-responsive polymers through a [2 + 2] cycloaddition [15,16]. These properties were used by some authors to create drug delivery systems based on light cross-linkable and pH de-cross-linkable micelles [17]. The incorporation of coumarin groups on linear silicone polymer backbones created silicone polymeric networks that exhibit thermoplastic elastomeric properties in the absence of covalent cross-links whose properties depend on the coumarin concentration [18]. From the published results we can summarize that the derivatization of the coumarin ring for incorporation into polymeric architectures have been attempted at the hydroxyl or amino function at the 7-position, or from the carboxylic derivative at the 3-position. The attachment of an acrylic or vinylic subunit necessary to the polymerization is usually accomplished through direct binding to these positions, or using a linker [19]. It is already well established that the absorption and the emission behavior of coumarins can be tuned depending on their functionalization. Our previous work [20,21] on coumarins demonstrated that these molecules can be easily adjusted to incorporate substituents that allow increasing the conjugation at the 3-position with a desirable red-shift of the absorption maxima. This effect is enhanced by the presence of electrodonating groups at the 7-position. When this is a hydroxyl group, the compound also presents chemiluminiscence [22], which can be an advantage given its applicability as carriers or probes for biological or in vitro detection. Given our experience in the synthesis of 3-vinylcoumarins [20,23] with enhanced strong fluorescence properties, our search for new polymeric materials is now turned on to this less-explored class of coumarin dyes. The vinyl functional group at the 3-position was never before studied as substrate to this type of reaction and appears very promising to study the incorporation of these compounds into polymeric chains. The aim of this study is the incorporation of 3-vinylcoumarins into co-polymers of styrene and methyl acrylate and spectroscopic characterization of the synthesized co-polymers.

## 2. Experimental Section

### 2.1. Materials and Methods

All reagents were obtained commercially from Sigma-Aldrich, Barcelona, Spain, and used without further purification. The solvents used were dried using current laboratory techniques. TLC was carried out on aluminum-backed Kieselgel 60 F254 silica gel plates (Merck, Kenilworth, NJ, USA). Plates were visualized by ultraviolet (UV) light (254 and/or 366 nm). Column chromatography was carried out on silica gel Kieselgel 60 (Merck), 70–230 mesh particle size as stationary phase. ^1^H and ^13^C nuclear magnetic resonance (NMR) spectra were recorded on a Bruker ARX400 (Karlsruhe, Germany) at 400 and 100 MHz, respectively. Fourier Transform-Infrared Spectroscopy (FTIR) was performed on the Perkin Elmer Two Spectrum spectrophotometer (Perkin Elmer, Boston, MA, USA) in support of KBr pellets, UATR or NaCl discs. MALDI-TOF-MS (Matrix Assisted Laser Desorption Ionization-Time Of Flight-Mass Spectrometry) mass spectra of the co-oligomers were run on a Voyager-DE PRO Workstation mass spectrometer (Applied Biosystems, Foster City, CA, USA), at REQUIMTE/FCT/Portugal in positive reflector mode (unless otherwise noted) or using a Bruker Daltonics UltrafleXtreme MALDITOF/TOF (Karlsruhe, Germany) at CEMUP/Porto/Portugal, being the matrix indicated case by case. ESI-MS (Electrospray ionization- Mass spectrometry) was performed in a LTQ Orbitrap XLTM mass spectrometer (Thermo Fischer Scientific, Bremen, Germany) controlled by LTQ Tune Plus 2.5.5 and Xcalibur 2.1.0. The capillary voltage of the electrospray ionization (ESI) was set to 3100 V. The capillary temperature was 275 °C. The sheath gas flow rate (nitrogen) was set to 5 (arbitrary unit as provided by the software settings). The capillary voltage was 36 V and the tube lens voltage 110 V. The description of these spectra follows the format: mass (*m*/*z*); attribution in the molecule. The designation [M + H]^+^ always corresponds to the molecular ion of interest.

3-Vinylcoumarin (**1**) and 7-methoxy-3-vinylcoumarin (**2**) were synthesized according to a previously published procedure [20].

### 2.2. Synthesis of 7-Hydroxy-3-Vinylcoumarin *(**3**)*

7-Hydroxy-3-vinylcoumarin (**3**) was synthetized according to a literature procedure for similar coumarines [20]. To a solution of *N*,*N*’-dicyclohexylcarbodiimide (3.72 g, 18.1 mmol, 2.5 eq) in dry dichloromethane was added 3-butenoic acid (1.54 mL, 18.1 mmol, 2.5 eq). After stirring for one hour under a nitrogen atmosphere, 2,3-dihydroxybenzaldehyde (1.0 g, 7,24 mmol) and 4-dimethylaminopyridine (0.22 g, 1.81 mmol, 0.25 eq) were added. The reaction mixture was stirred and after 3 h a total consumption of the aldehyde was verified by TLC. The reaction was filtered, and to the filtrate was added Cs_2_CO_3_ (2.83 g, 8.688 mmol, 1.2 eq). The reaction was followed by TLC (hexane/ethyl acetate (7:3)) and total conversion to the coumarin was observed after 24 h. To the reaction mixture was added a saturated solution of NaHCO_3_ (50 mL), and the mixture was left under vigorous stirring for 24 h. After 24 h the reaction was acidified with 1 M HCl solution and the reactional mixture was washed with DCM (3 × 100 mL) until all the compound was removed from the aqueous phase. Purification by chromatography column using hexane/ethyl acetate (7:3) as eluent allowed **3** to be obtained as a yellow solid (1.09 g, 80%). IV (KBr) *ν*_max_ (cm^−1^): 3237 (OH), 1688 (C=O), 1612 (C=C). ^1^H-RMN (CD_3_CN) δ (ppm): 8.07 (1H, s, ArH4), 7.53 (1H, d, *J* = 8.5 Hz, ArH5), 6.8 (1H, dd, *J* = 2.2 e 8.5 Hz, H6), 6.71 (1H, d, *J* = 2 Hz, H8), 6.62 (1H, dd, *J* = 11.4 e 17.6 Hz, Hα), 6.1 (1H, dd, *J* = 1.4 e 17.6 Hz, Hβ’), 5.38 (1H, dd, *J* = 1.3 e 11.4 Hz, Hβ). ^13^C-RMN (CD_3_CN) δ (ppm): 161.3 (C=O/ArC9/ArC7), 159.8 (ArC9/ArC7), 154.6 (ArC9/ArC7), 139.6 (ArC4), 130.9 (ArC5/CH vinyl), 129.9 (ArC5/CH vinyl), 117.3 (ArC10/ArC3/CH2 vinyl), 113.6 (ArC10/ArC3/CH2 vinyl), 111.7 (ArC10/ArC3/CH2 vinyl), 101.9 (ArC8). ESI-MS/MS (189.05): *m*/*z* 161.06 [MH–CO]^+^, 145.06 [MH–CO_2_]^+^, 133.06 [MH–CO–CH=CH_2_]^+^. HRMS-ESI(+) calculated for C_11_H_9_O_3_ [M + H]^+^ 189.05462 found 189.05442.

### 2.3. Synthesis of Co-Oligomers

*Synthesis of the co-oligomer of styrene with 3-vinylcoumarin* (**1b**): To a solution of 3-vinylcoumarin **1** (0.112 g, 0.649 mmol, 1 eq) in 5 mL of acetonitrile (dry and deoxygenated) was added styrene (0.38 mL, 3.25 mmol, 5 eq) and 2,2-azobis(isobutyronitrile) (AIBN) (0.061 g, 0.371 mmol, 0.57 eq) which was added at once and the solution was refluxed. Since after 24 h the monomers were still identified by TLC in solution, 0.03 g more AIBN was added, and after 48 h the procedure was repeated with more 0.03 g of AIBN being added. The reaction took 72 h to consume both monomers. The reaction was followed by TLC (hexane/ethyl acetate, 7:3). The acetonitrile was evaporated to dryness and the co-polymer was precipitated by dissolving the residue in methanol. A yellowish-brown solid (0.042 g) was obtained. ${\bar{M}}_{w}$ = 880, ${\bar{M}}_{n}$ = 808 and *D* = 1.09 as calculated from MALDI-TOF (matrix: dithranol + Ag). IV (KBr) *ν*_max_ (cm^−1^): 1717 (C=O), 1605 (C=C). ^1^H-RMN (CDCl_3_) δ (ppm): 7.49–6.96 (bs, ArH), 1.73–1.55 (bs, CH), 1.25 (CH2).

*Synthesis of co-oligomer of styrene with 7-methoxy-3-vinylcoumarin* (**2b**): To a solution of 7-methoxy-3-vinylcoumarin **2** (0.075 g, 0.371 mmol, 1 eq) in 7 mL of acetonitrile (dry and deoxygenated), styrene (0.21 mL, 1.855 mmol, 5 eq) and 2,2-azobis(isobutyronitrile) (AIBN) (0.012 g, 0.073 mmol, 0.2 eq) were added gradually through an automatic injector during 3 h under reflux. After 24 h, 0.006 g more AIBN (0.1 eq) was added. The reaction took 72 h to consume both monomers and was followed by TLC (hexane/ethyl acetate, 7:3). The acetonitrile was evaporated to dryness and the co-polymer precipitated by dissolving the residue in chloroform followed by dropwise addition of cold hexane. A yellow solid was obtained (0.061 g). (KBr) *ν*_max_ (cm^−1^): 1720 (C=O), 1613 (C=C). ^1^H-RMN (CDCl_3_) δ (ppm): 7.37 (bs, ArH), 6.82 (bs, ArH), 3.87 (bs, OCH3), 2.06–1.61 (bs, CH), 1.26 (bs, CH2). ^13^C-RMN (CDCl_3_) δ (ppm): 162.6 (C=O), 161.4 (C7-O), 154.9 (C9-O), 140.6 (CH2-ArC styrene), 139.8 (ArC4 coumarin), 133.3 (ArC3 coumarin), 130.1–126.1 (ArC styrene, ArC5 coumarin), 112.9–112.7 (ArC6, ArC10 coumarin), 100.5 (ArC8 coumarin), 74.7 (OCH3), 70.3 (OCH3), 68.1 (OCH3), 65.3 (OCH3), 55.8 (OCH3), 40.5 (CH, CH2).

*Synthesis of co-oligomer of methyl acrylate with 7-methoxy-3-vinylcoumarin* (**2a**): To a solution of 7-methoxy-3-vinylcoumarin **2** (0.075 g, 0.347 mmol, 1 eq) in 5 mL of acetonitrile (dry and deoxygenated), methyl acrylate (0.167 mL, 1.855 mmol, 5 eq) and 2,2-azobis(isobutyronitrile) (AIBN) (0.0609 g, 0.371 mmol, 0.1 eq) were added; half was added in the beginning of the reaction, and the other half 24 h after. The reaction was maintained at reflux for 72 h until both monomers were consumed as followed by TLC (hexane/ethyl acetate, 7:3). The acetonitrile was taken to dryness and the co-polymer was precipitated by dissolving the residue in methanol and dropwise addition of water. A brown solid was obtained (0.058 g). ${\bar{M}}_{w}$ = 1279, ${\bar{M}}_{n}$ = 1172 and *D* = 1.08 as calculated from MALDI-TOF (matrix: 2,4-dihydroxybenzoic acid (DHB)). IV (KBr) *ν*_max_ (cm^−1^): 1729 (C=O), 1612 (C=C). ^1^H-RMN (CDCl_3_) δ (ppm): 7.82 (bs, ArH4), 7.4–7.38 (bs, ArH5), 6.91–6.79 (bs, ArH6/ArH8), 3.85 (bs, OCH3 coumarin), 3.64 (bs, OCH3 ethyl acrylate), 2.29 (bs, CHCOOMe), 1.68 (bs, CH2), 1.28–1.24 (bs, CH). ^13^C-RMN (CDCl_3_) δ (ppm): 174.9 (C=O methyl acrylate), 162.6 (C=O coumarin), 161.4 (C7-O), 154.9 (C9-O), 139.8 (ArC4), 133.3 (ArC3), 129 (ArC5), 113.1 (ArC10), 112.7 (ArC6), 100.4 (ArC8), 72.6 (OCH3), 70.3 (OCH3), 65.3 (OCH3), 64.5 (OCH3), 55.8 (OCH3 coumarin), 51.8 (OCH3 methyl acrylate), 41.2 (CH, CH2).

*Synthesis of co-oligomer of methyl acrylate with 7-hydroxy-3-vinylcoumarin* (**3a**): To a solution of 7-hydroxy-3-vinylcoumarin **3** (0.059 g, 0.3138 mmol, 1 eq) in 5 mL of acetonitrile (dry and deoxygenated), methyl acrylate (0.14 mL, 1.569 mmol, 5 eq) and 2,2-azobis (isobutyronitrile) (AIBN) (0.01 g, 0.06276 mmol, 0.2 eq, in one portion) were added. Each 24 h more AIBN was added, to a total of 0.02 g (0.1218 mmol, 0.4 eq). The reaction took 72 h to consume both monomers, as followed by TLC (hexane/ethyl acetate, 7:3). The acetonitrile was taken to dryness and the co-polymer was precipitated by dissolving the residue in diethyl ether. A brown solid was obtained (0.024 g). ${\bar{M}}_{w}$ = 1062, ${\bar{M}}_{n}$ = 1117, and *D* 1.05 as calculated from MALDI-TOF (matrix: *trans*-2-[3-(4-*tert*-butylphenyl)-2-methyl-2-propenylidene]malononitrile (DCTB)). IV (KBr) *ν*_max_ (cm^−1^): 3331 (OH), 1739 (C=O), 1611 (C=C). ^1^H-RMN (CD_3_CN) δ (ppm): 7.39–7.16 (bs, ArH5/ArH4), 6.75 (bs, ArH6/ArH8), 3.61 (bs, OCH3), 2.26 (bs, CHCOOMe), 1.81–1.64 (bs, CH2), 1.32–1.18 (bs, CH2). ^13^C-RMN (CD_3_CN) δ (ppm): 174.9 (C=O methyl acrylate), 160.2 (C=O coumarin), 157.3 (C7-O), 154.7 (C9-O), 141.4 (ArC4), 129 (ArC3), 124.5 (ArC10), 123.7 (ArC5), 113 (ArC6), 102.1 (ArC8), 51.4 (OCH3 methyl acrylate), 41.2 (CH, CH2).

*Synthesis of co-polymers of methyl acrylate with 3-vinylcoumarin* (**1a**): To a solution of 3-vinylcoumarin **1** (0.25 g, 1.45 mmol, 1 eq) in 10 mL of acetonitrile (dry and deoxygenated), methyl acrylate (0.65 mL, 7.25 mmol, 5 eq) and 2,2-azobis (isobutyronitrile) (AIBN) (0.1372 g, 0.84 mmol, 0.58 eq) were added, half in the beginning of the reaction and the other half after 7 h. The reaction took 24 h to consume both monomers, as followed by TLC (hexane/ethyl acetate, 7:3). The acetonitrile was taken to dryness and the co-polymer was precipitated by dissolving the residue in methanol. A brown solid was obtained (0.275 g). *M*_w_ = 1386.7 a.m.u. *M*_n_ = 1500.7 a.m.u. PD = 0.92 as calculated from MALDI-TOF (matrix: DCTB). IV (NaCl) *ν*_max_ (cm^−1^): 1739 (C=O), 1610 (C=C). ^1^H-RMN (CDCl_3_) δ (ppm): 7.62–7.29 (bs, ArH coumarin), 3.65 (bs, OCH3), 2.3 (bs, CHCOOMe), 1.93 (bs, CH2), 1.67 (bs, CH2). ^13^C-RMN (CD_3_CN) δ (ppm): 174.9 (C=O methyl acrylate), 160.7 (C=O coumarin), 153 (C9-O), 131.5 (ArC4), 127.9 (ArC3), 124.7 (ArC5), 124.4 (ArC6), 119.1 (ArC10), 116.5 (ArC8), 51.8 (OCH3 methyl acrylate), 41.4–41.1 (CH, CH2).

### 2.4. Spectrosocpy and Fluorescence Quantum Yields

UV-Vis absorption spectra were recorded on a Thermo Corp. (Waltham, MA, USA) spectrophotometer, Helius γ, on quartz cells. Molar absorptivity coefficients of **3** were determined from a 1.60 × 10^−4^ M solution of this compound in ethanol; for the basic form, the solution was basified by addition of two drops of an 0.5 M ethanolic solution of NaOH. The Emission and excitation spectra were made on a SPEX Fluorolog-3 Model FL3-22 or on a Perkin-Elmer LS45 spectrofluorimeter (Perkin Elmer, Boston, MA, USA) using quartz cells. The fluorescence quantum yields were determined using 7-(*N*,*N*-diethylamine)-4-methylcoumarin (Ф_f_ = 73%) in ethanol as reference [24].

## 3. Results and Discussion

### 3.1. Synthesis

**Scheme 1:**
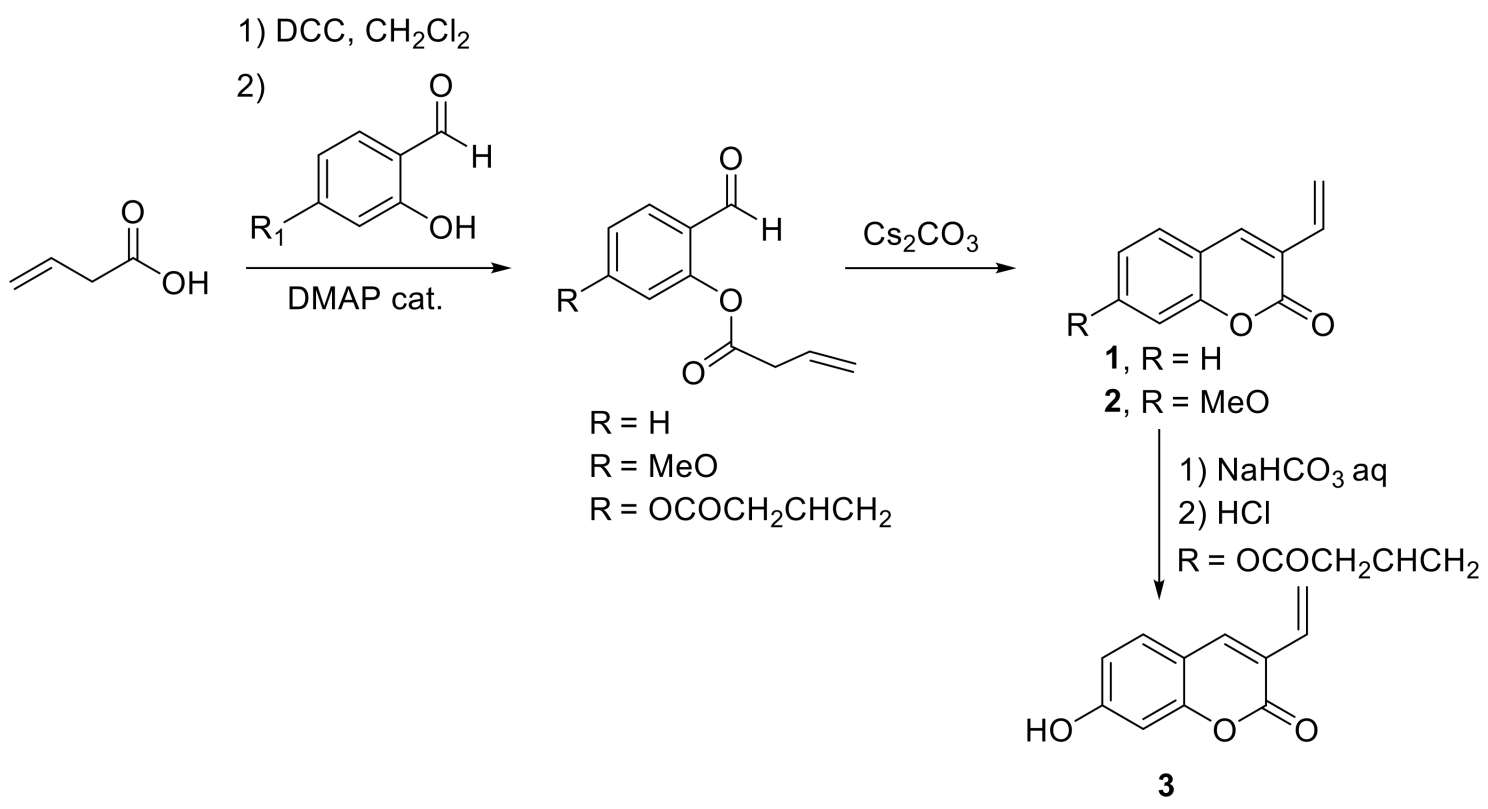
Synthetic approach to the synthesis of 3-vinyl coumarins (**1**–**3**) following a one-pot procedure.

For the synthesis of the 3-vinylcoumarin monomers (**1**–**3**) our previously described procedure was used [20], as presented in Scheme 1. The 7-hydroxy-3-vinylcoumarin **3** described here for the first time was achieved starting from 2,4-dihydroxybenzaldehyde and 2 equivalents of 3-butenoic acid, followed by in situ hydrolysis.

**Scheme 2:**
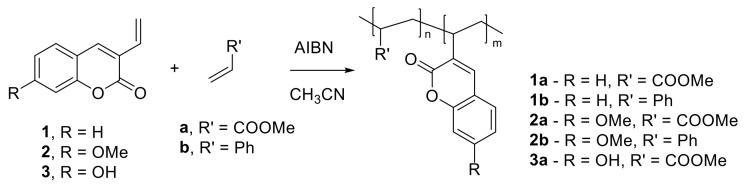
Polymerization reaction between the 3-vinylcoumarin monomers (**1**–**3**) and styrene or methyl acrylate.

The unsaturation at the 3-positon through the vinyl group was used to endeavor the co-polymerization reactions with methyl acrylate or styrene, using AIBN as a radical initiator to attain the polymers (Scheme 2). The amount of AIBN used stayed between 5% and 20% of the total amount of monomers used. The addition of AIBN was made with a syringe pump; if the reaction evolution seemed to be stopped and the presence of monomers were still identified on the reaction medium, more AIBN was added.

### 3.2. FTIR, NMR and Mass Spectrometry Characterization of Co-Oligomer

All the synthesized co-oligomers were analyzed by FTIR and ^1^H and ^13^C NMR spectroscopy. Applying MALDI-TOF or ESI mass spectrometry techniques we attempt structure analysis and polymer identification, since the mass spectrum of a polymer possesses characteristic fragment peaks that are peculiar to a polymer structure and correspond to the monomers used. For the calculation of the number-average molar mass (${\bar{M}}_{n})$, average molar mass (${\bar{M}}_{w}$) and Polydispersity index (*D*) Equations (1)–(3) were used, respectively [25]. Characteristic to all structures are the broad signals (bs) presented by the NMR spectra (see Supporting Information). For **1b**, a co-oligomer of styrene and coumarin **1**, the signals are slightly deshielded in relation to polystyrene which is due to the presence of the coumarin ring and corroborated by the band in the FTIR spectra at 1717 cm^−1^ for the carbonyl group. The mass spectrum presents the characteristic Gaussian distribution of masses for a polymeric structure. For co-polymer **1b** the values are respectively ${\bar{M}}_{n}$ 808, ${\bar{M}}_{w}$ 880 and D 1.09. As an example, ion *m*/*z* 1143 (see Supporting Information) can be attributed to five molecules of styrene, three of coumarin **1**, a terminal isobutyronitrile and a potassium ion.

(1) $${\bar{M}}_{n}=\left(\Sigma m_{i}N_{i}\right)/\left(\Sigma N_{i}\right)$$

(2) $${\bar{M}}_{w}\;=\left(\Sigma m_{i}^{2}N_{i}\right)/\left(\Sigma m_{i}N_{i}\right)$$

(3) $$D={\bar{M}}_{w}\;/{\bar{M}}_{n}$$

where *N*_i_ is the number of molecules of mass *m*_i_.

For the co-oligomer **2b**, the presence of the coumarin moiety **2** is clearly identified by the ^1^H NMR spectra by the broad signal at 3.8 ppm and the shielding of the aromatic protons *ortho* to the methoxy group at 6.8 ppm (see Supporting Information). As expected, the presence of the coumarin ring is also verified by the signals at 161 and 162 ppm on the ^13^C NMR spectra and on the FTIR by the band at 1720 cm^−1^ due to the carbonyl group.

**Figure 1:**
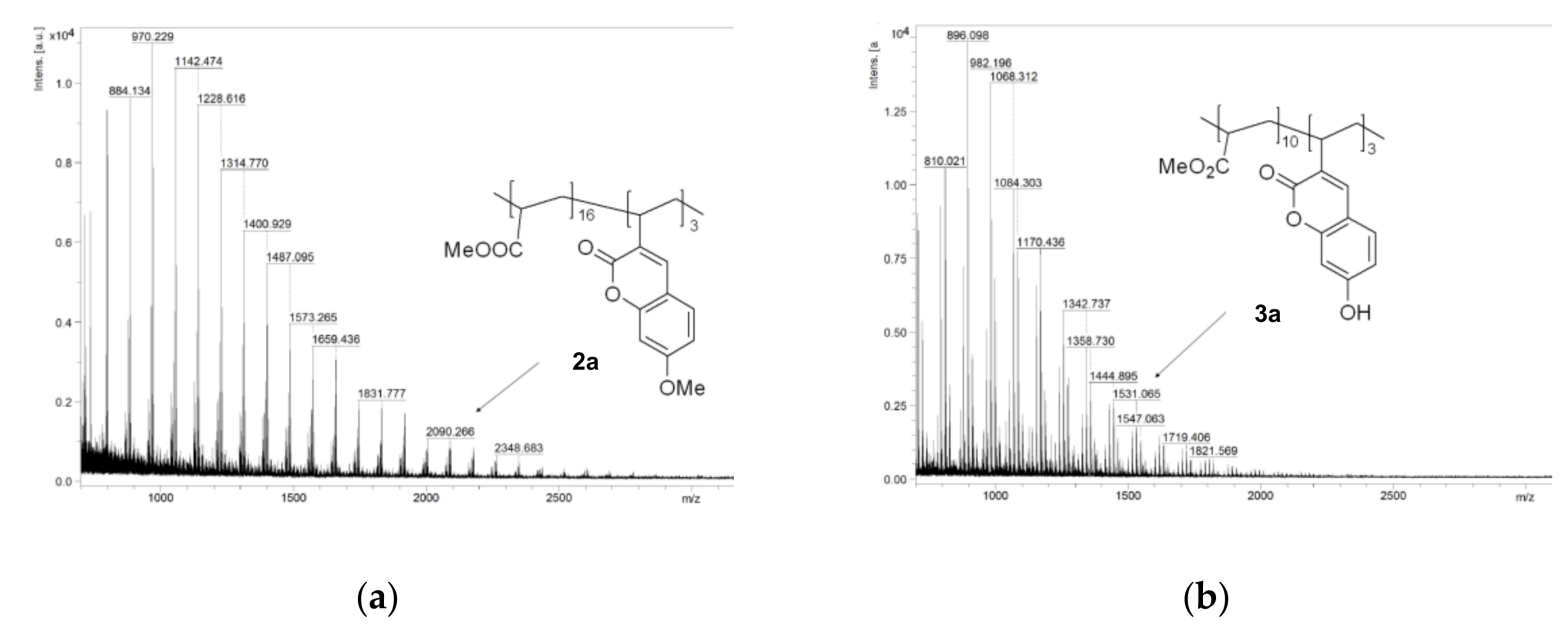
MALDI-TOF mass spectra of: (**a**) co-oligomer **2a** in 2,4-dihydroxybenzoic acid (DHB) matrix (inset assignment of a possible constitution of ion *m*/*z* 2091) and; (**b**) co-oligomer **3a** in *trans*-2-[3-(4-*tert*-butylphenyl)-2-methyl-2-propenylidene]malononitrile (DCTB) matrix (inset assignment of a possible constitution of ion *m*/*z* 1531). The isobutyronitrile terminal and the potassium ion are omitted.

As for the co-oligomer **2a** from de co-polymerization reaction between the 7-methoxy-3-vinycoumarin (**2**) and methyl acrylate the presence of the two methoxy groups are clearly identified by the broad signals at 3.6 and 3.8 ppm on the ^1^H NMR. The MALDI-TOF spectra showing a characteristic Gaussian distribution (Figure 1) allowed us to calculate the number-average molar mass (${\bar{M}}_{n})$ average molar mass, ${\bar{M}}_{w}$ and Polydispersity index (*D*), which are ${\bar{M}}_{n}$ 975, ${\bar{M}}_{w}$ 899 and *D* 1.08 respectively. For example, ion *m*/*z* 2091 can be attributed to sixteen molecules of methyl acrylate, three of coumarin **2**, a terminal isobutyronitrile and a potassium ion. We cannot exclude that some hydrolysis of the ester group from methyl acrylate may have occurred due to the broad band at 3455 cm^−1^ on the FTIR spectra.

**Figure 2:**
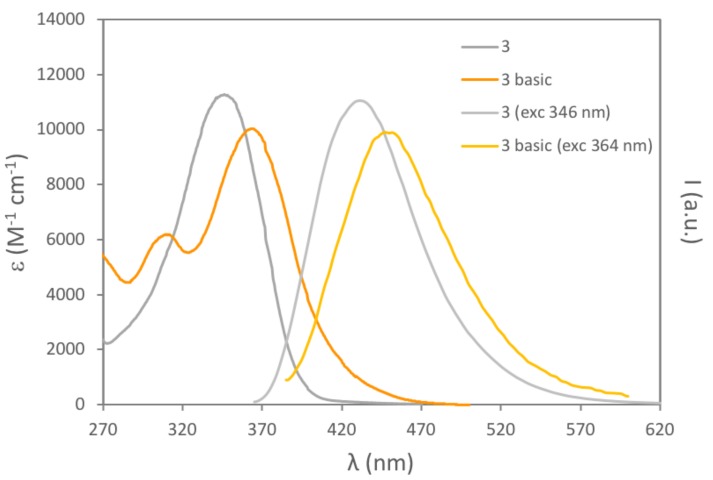
Absorption and emission spectra for 7-hydroxi-3-vinylcoumarin (**3**) in ethanol and basified ethanol (1M NaOH).

As for the co-oligomer **3a**, the presence of broad signals on the ^1^H NMR spectra between 6.5 and 7.5 ppm are indicative of the presence of the coumarin moiety and the broad singlet at 3.6 ppm is due to the methoxy group from methyl acrylate monomer. The coumarin moiety is clearly identified on the FTIR spectra by a broad band at 3330 cm^−1^ for the hydroxyl group and a broad band centered at 1739 cm^−1^ for both carbonyl groups. The MALDI-TOF spectra analysis allowed to determine ${\bar{M}}_{n}$ 1062, ${\bar{M}}_{w}$ 1117 and D 1.05. For example, ion *m*/*z* 1531 (Figure 2) can be attributed to ten molecules of methyl acrylate, three of coumarin **3**, a terminal isobutyronitrile and a potassium ion.

For co-oligomer **1a**, although the information retrieved from FTIR and ^1^H NMR spectroscopy is indicative for the presence of a co-oligomeric molecule, no information could be obtained about its constitution from the MALDI-TOF mass spectra, since the molecular mass of both monomers are multiple (86 Da vs. 172 Da). The MALDI-TOF spectra analysis allowed to determine the ${\bar{M}}_{w}$ = 1386.7 a.m.u. ${\bar{M}}_{n}$ = 1500.7 a.m.u. and *D* = 0.92.

### 3.3. Spectroscopic and Photophysical Studies

The spectroscopic behavior of **3** was evaluated in ethanol under neutral and basic conditions (Figure 2). The acidic phenol species presents a maximum at 346 nm that compares with the values of 327 nm for 7-hydroxycoumarin and with 335 and 343 nm, for coumarins **1** and **2**, respectively (Table 1). As expected, the presence of the vinyl group at the 3-position causes a bathochromic shift on the UV absorption maximum relative to 7-hydroxycoumarin as a result of the extension of the conjugated system. On the other hand, the presence of the electron donating OH group in position 7 of **3** increases the charge transfer character of the transition in comparison with compound **1** and makes it comparable with compound **2**, which contains a similar electron donor group (OMe). The phenolate species that forms in basic solution (EtOH/NaOH) has a maximum at 366 nm, red-shifted relative to the acidic species as usually observed for phenols and in particular in hydroxycoumarins [26,27,28]. The emission of **3** peaks at 431 nm with a fluorescence quantum yield of 70% while that of the basic form occurs at 447 nm with a quantum yield of 80%.

**Figure 3:**
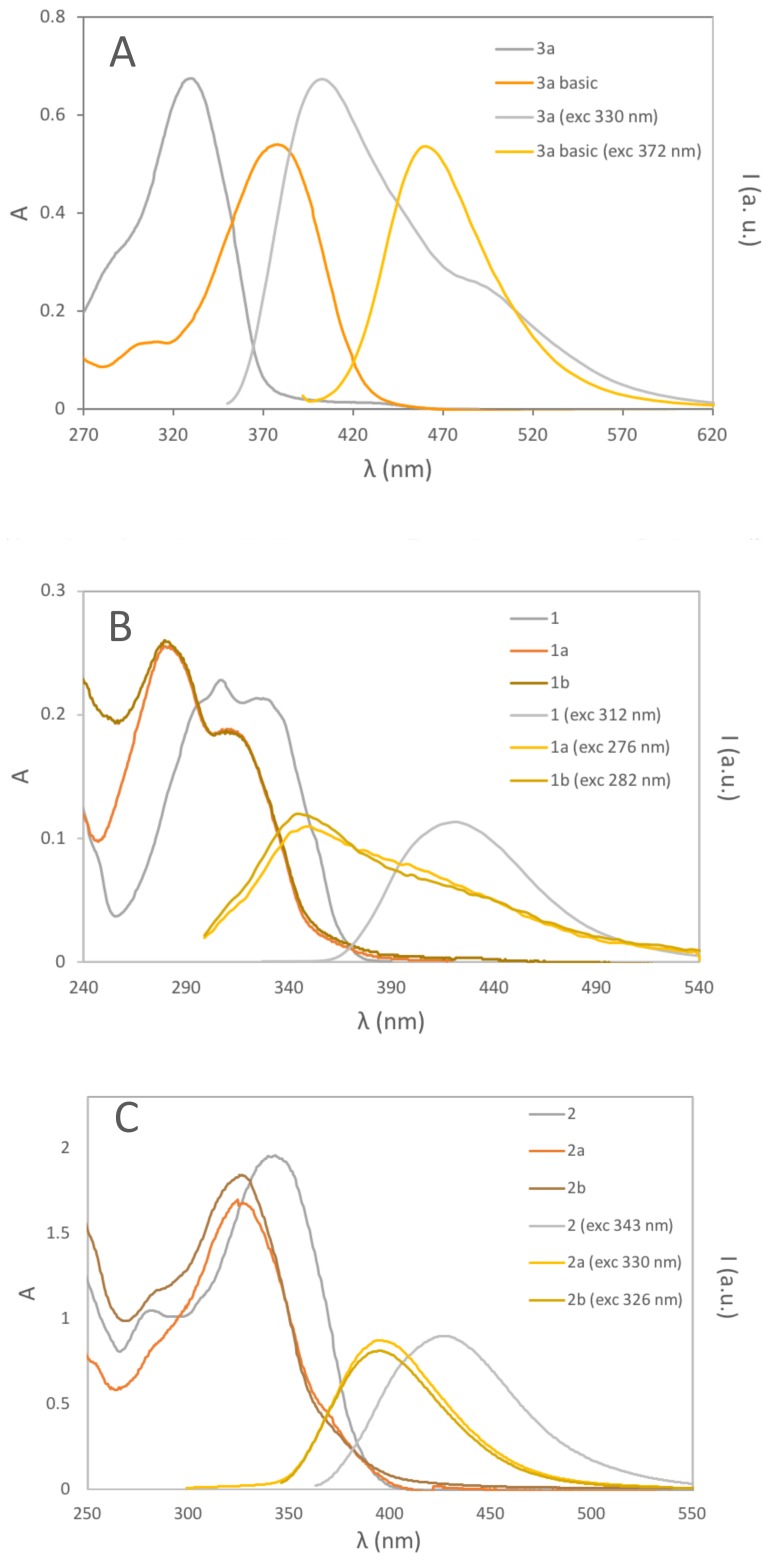
Absorption and emission spectra in ethanol for methyl acrylate co-oligomer **3a** under neutral and basic conditions (**A**); coumarin **1** and corresponding styrene **1b** and methyl acrylate **1a** co-oligomers (**B**); coumarin **2** and corresponding styrene **2b** and methyl acrylate **2a** co-oligomers (**C**).

Upon polymerization, the vinyl group of the coumarin monomers no longer exists and this is reflected in the absorption spectra. Table 1 collects the spectroscopic and photophysical data of the monomers and polymers. For instance, in the case of **3a** (co-oligomer between methyl acrylate and **3**) the value of λ_max_ decreases from 346 nm in **3** to 330 nm in **3a** (Figure 3A). This also reflects in the emission maxima: 431 nm in the monomer vs. 403 nm in the co-oligomer derived methyl acrylate, giving rise to similar Stokes shifts. Similar results were observed for coumarin **1** and its co-oligomers **1a** and **1b** and for coumarin **2** and its co-oligomers **2a** and **2b**, cf. Table 1 and Figure 3B,C.

The fluorescence quantum yields of the co-oligomers **2a**, **2b** and **3a**, derived from monomers **2** and **3**, are close to those of the model coumarins lacking the vinyl group: 7-hydroxycoumarin has Ф_f_ = 0.11 in ethanol [29] and 7-methoxycoumarin has Ф_f_ = 0.05 in methanol [30], which are significantly lower than those of the original vinylcoumarins **2** and **3**.

## 4. Conclusions

The synthesis of 3-vinylcoumarins and, in particular, of the new derivative 7-hydroxy-3-vinylcoumarin (**3**), allowed us to obtain monomers for further incorporation into polymeric backbones based on styrene and methyl acrylate using AIBN as a radical initiator. The structural characterization by NMR, MALDI-TOF-MS and UV-Vis absorption and emission spectroscopy clearly demonstrate the incorporation of the coumarin monomers into the polymeric chain. Measurements of the fluorescence quantum yields of the monomers and polymers show a decrease of Ф_f_ in the polymers upon loss of the vinyl group. The applicability of the synthesized polymers will be the target of future studies.

## Figures and Tables

**Table 1:** Spectrosocopic and photophysical data of the monomers and polymers in ethanol.

Compound	λ_max_/nm (ε/M^−1^cm^−1^)	λ_em_/nm	Ф_f_
**1**	312, 335	424	0.08 ^1^ [20]
**2**	343	430	0.99 ^1^ [20]
**3**	346 (11,000)	431	0.70
**3-basic solution ^2^**	364 (9920)	447	0.80
**1a**	276, 314 ^3^	352, 400 ^3^	0.01
**1b**	282, 320 ^3^	347, 410 ^3^	0.01
**2a**	330	394	0.16
**2b**	326	390	0.15
**3a**	330	403, 497 ^3^	0.11
**3a-basic solution ^2^**	372	460	0.16

^1^ In acetonitrile; ^2^ Ethanol basified with sodium hydroxide; ^3^ Shoulder.

## Supplementary Materials

The following are available online at http://www.mdpi.com/2073-4360/10/4/396/s1, Figures S1–S5: ^1^H, ^13^C NMR spectra, FTIR and Mass spectrum of 7-hydroxy-3-vinylcoumarin (**3**); Figures S6–S8: ^1^H NMR spectra, FTIR and Mass spectrum of the co-oligomer (**1b**); Figures S9–S12: ^1^H, ^13^C NMR spectra, FTIR and Mass spectrum of the co-oligomer (**2b**); Figures S13–S16: ^1^H, ^13^C NMR spectra, FTIR and Mass spectrum of the co-oligomer (**2a**); Figures S17–S20: ^1^H, ^13^C NMR spectra, FTIR and Mass spectrum of the co-oligomer (**3a**); Figures S21–S24: ^1^H, ^13^C NMR spectra, FTIR and Mass spectrum of the co-oligomer (**1a**).
