# Supplementary material for: Incorporation of Coumarin-Based Fluorescent Monomers into Co-Oligomeric Molecules
Source: Polymers (Basel). 2018 Apr 3;10(4):396. doi: 10.3390/polym10040396 (PMC6415208; doi:10.3390/polym10040396)
Supplement: Supplementary file 1 [file polymers-10-00396-s001.pdf]

Supporting Information

INCORPORATION OF COUMARIN-BASED FLUORESCENT MONOMERS INTO CO-OLIGOMERIC MOLECULES

Edgar Teixeira,[1] João C. Lima,[1] A. Jorge Parola[1,]* and Paula S. Branco[1,]*

[1] LAQV-REQUIMTE, Departamento de Química, Faculdade de Ciências e Tecnologia, UnFTIRersidade NOVA de Lisboa, 2829-516 Caparica, Portugal

***** Correspondence: ajp@fct.unl.pt; paula.branco@fct.unl.pt; Tel.: +351-212948300

Contents

^{1}H, ^{13}C NMR spectra, FTIR and Mass spectrum of 7-Hydroxy-3-vinylcoumarin (3) S2

^{1}H, ^{13}C NMR spectra, FTIR and Mass spectrum of the co-oligomers S4

Co-oligomer of styrene with 3-vinylcoumarin (**1b**) S4

Co-oligomer of styrene with 7-methoxy-3-vinylcoumarin (**2b**) S6

Co-oligomer of methyl acrylate with 7-methoxy-3-vinylcoumarin (**2a**) S8

Co-oligomer of methyl acrylate with 7-hydroxy-3-vinylcoumarin (**3a**)................................ S10

Co-oligomer of methyl acrylate with 3-vinylcoumarin (**1a**) S12

^{1}H, ^{13}C NMR spectra, FTIR and Mass spectrum of 7-Hydroxy-3-vinylcoumarin (3)

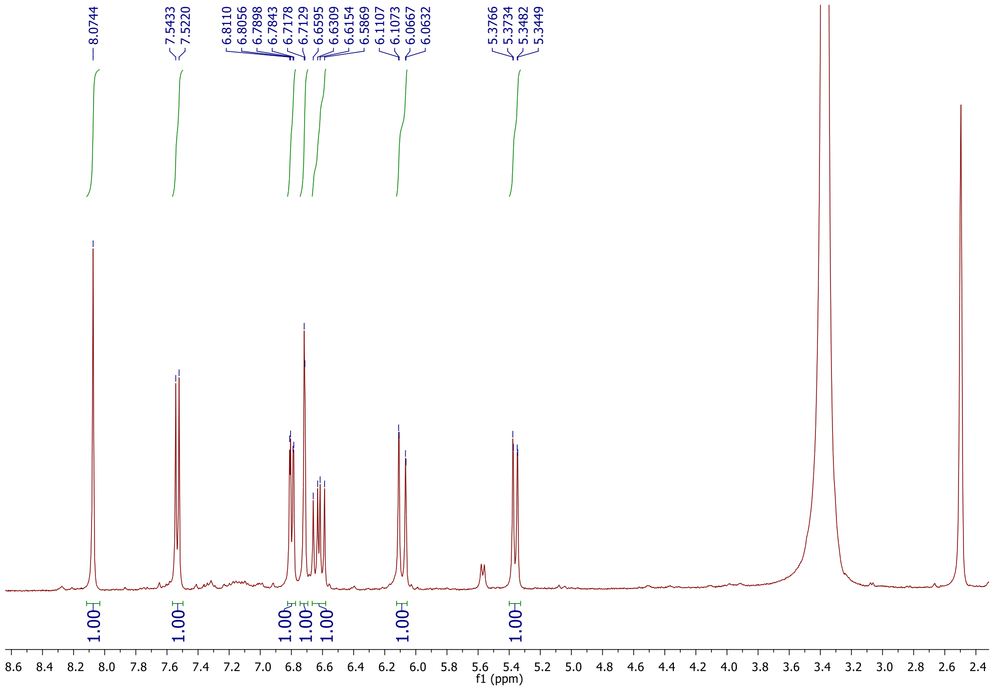

Figure S1 - ^{1}H (400 MHz, DMSO) of 7-hydroxy-3-vinylcoumarin (**3**).

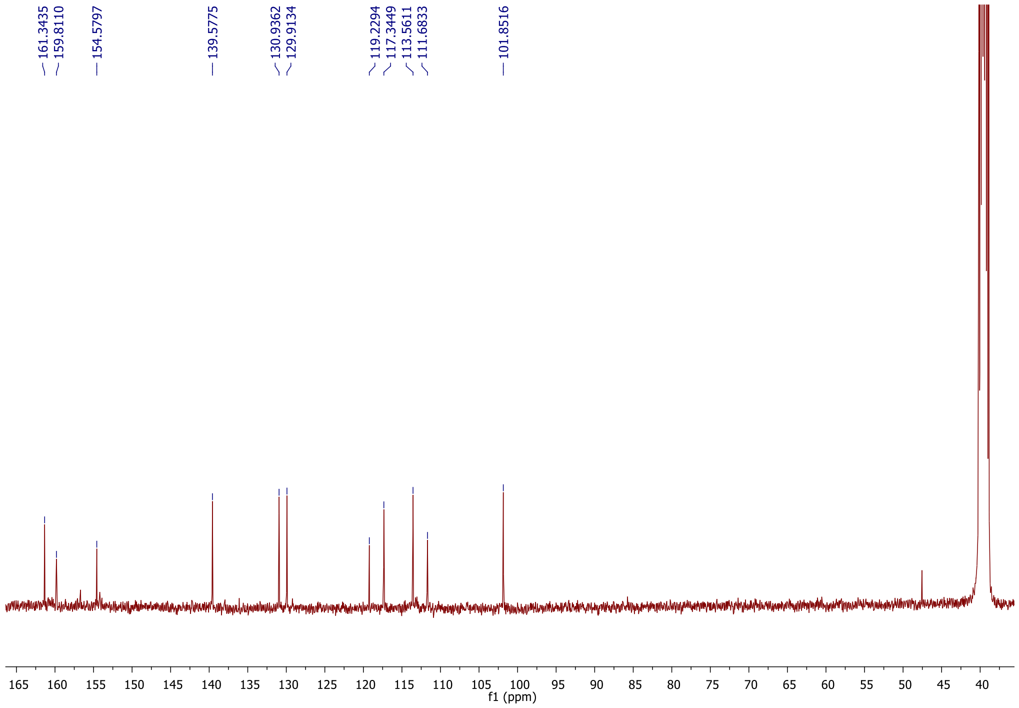

Figure S2 - ^{13}C (100 MHz, DMSO) of 7-hydroxy-3-vinylcoumarin (3).

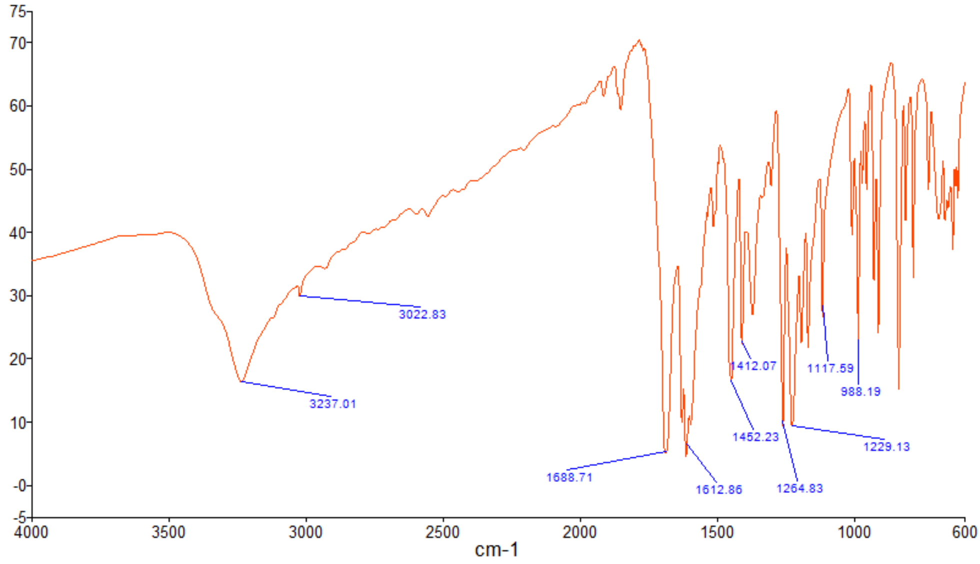

Figure S3 - FTIR (KBr) of compound 7-hydroxy-3-vinylcoumarin (**3**).

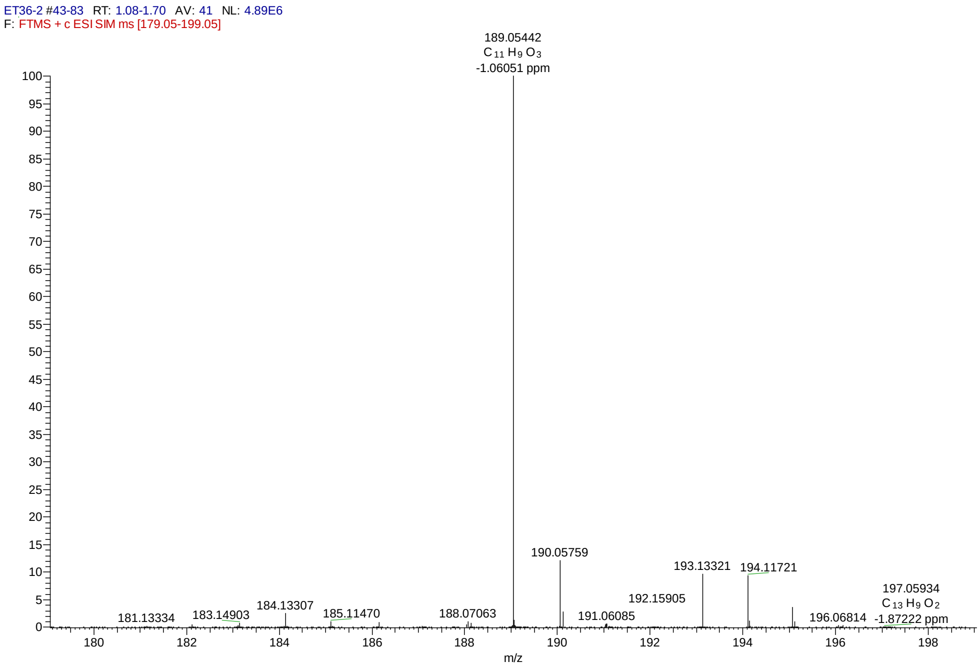

Figure S4 - HRMS-ESI (+) of 7-hydroxy-3-vinylcoumarin (**3**).

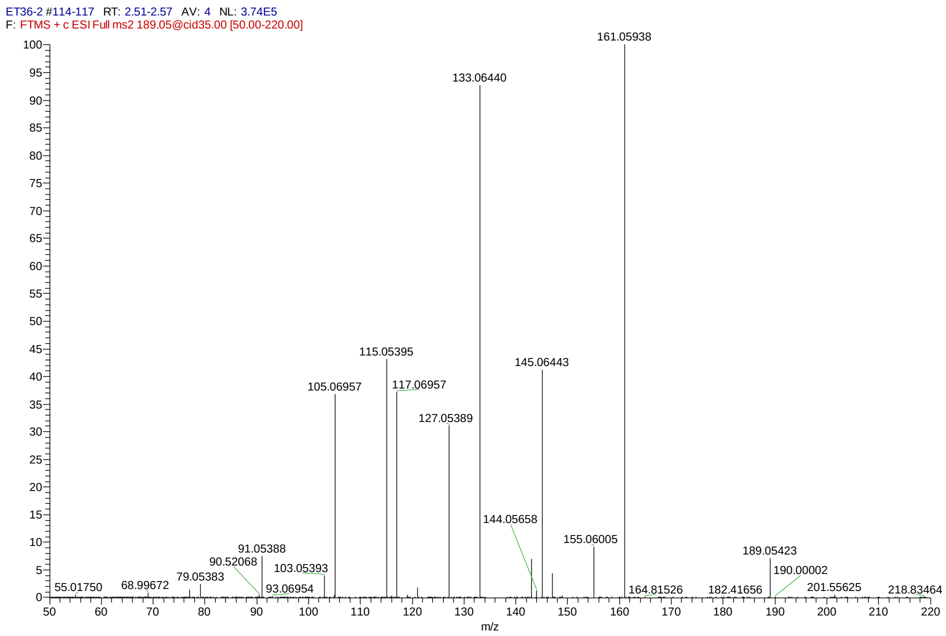

Figure S5 - ESI-MS/MS (189) spectrum of 7-hydroxy-3-vinylcoumarin (**3**).

^{1}H, ^{13}C NMR spectra, FTIR and Mass spectrum of the co-oligomers

Co-oligomer of styrene with 3-vinylcoumarin (**1b**)

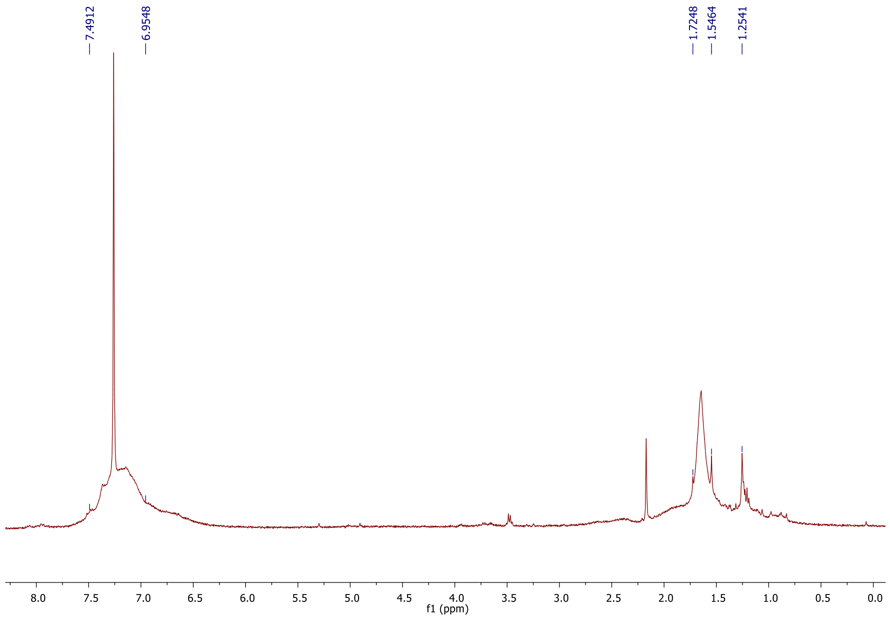

Figure S6 - ^{1}H (400 MHz, CDCl3) of co-oligomer **1b**.

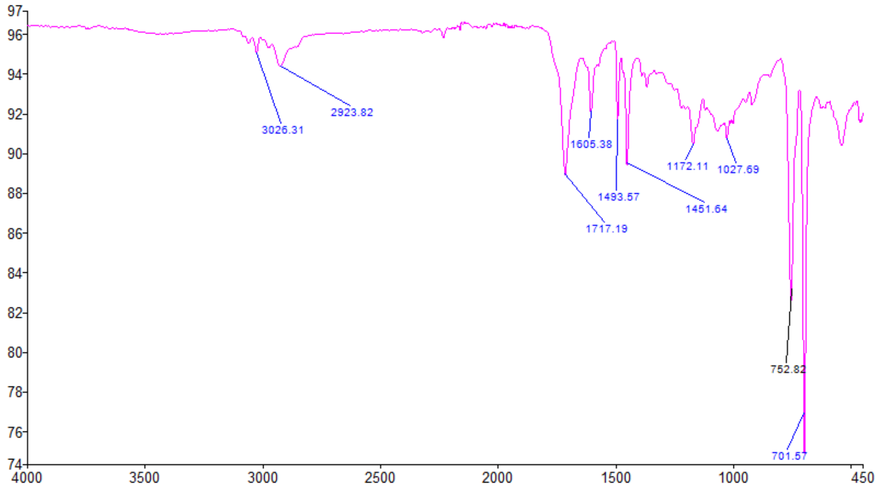

Figure S7 - FTIR (KBr) of co-oligomer **1b**.

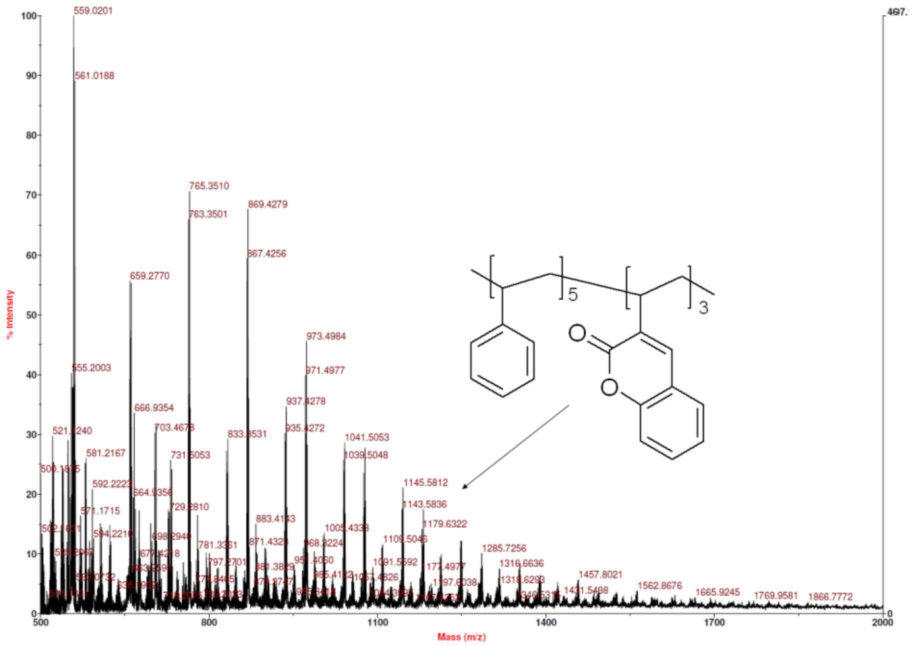

Figure S8 - MALDI-TOF Mass spectrum (matrix - dithranol + Ag) of co-oligomer **1b**. Assigned in the spectrum is the ion with m/z 1143 which can be attributed to five molecules of styrene, three of coumarin **1**, a terminal isobutyronitrile and a potassium ion.

Co-oligomer of styrene with 7-methoxy-3-vinylcoumarin (2b)

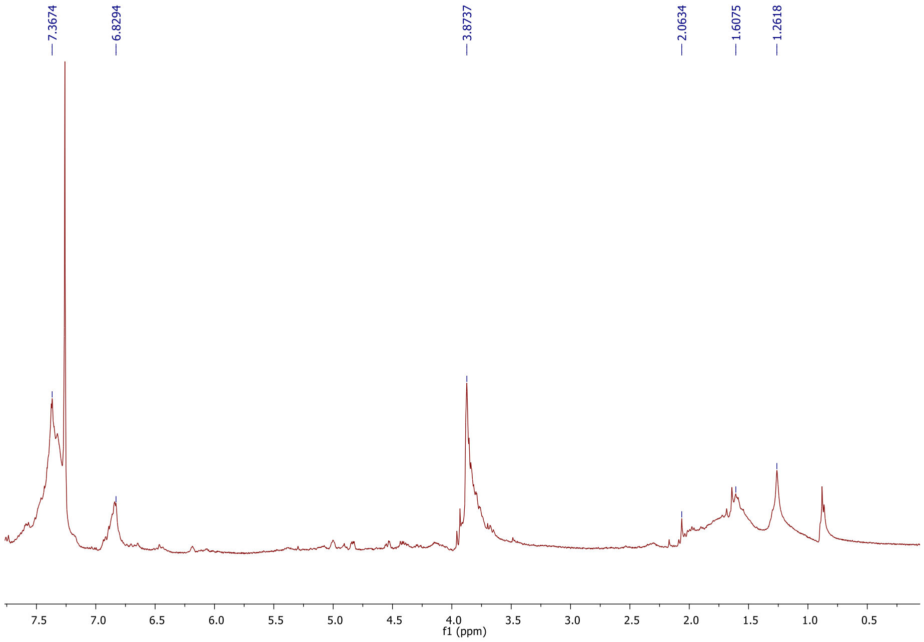

Figure S9 - ^{1}H (400 MHz, $CDCl_3$) of co-oligomer **2b**.

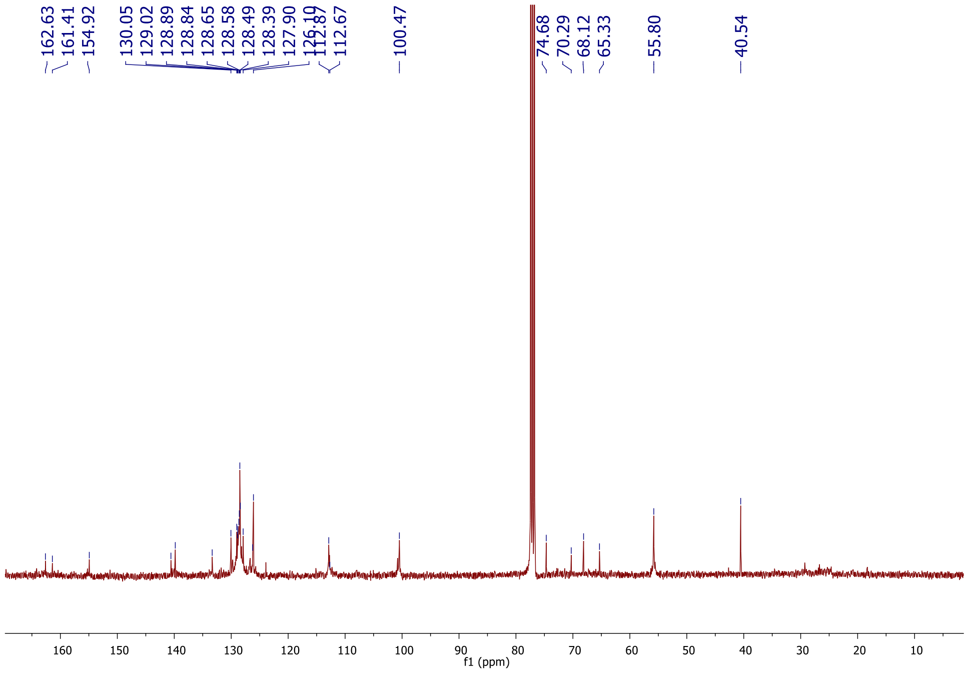

Figure S10 - ^{13}C (100 MHZ, $CDCl_3$) of co-oligomer **2b**.

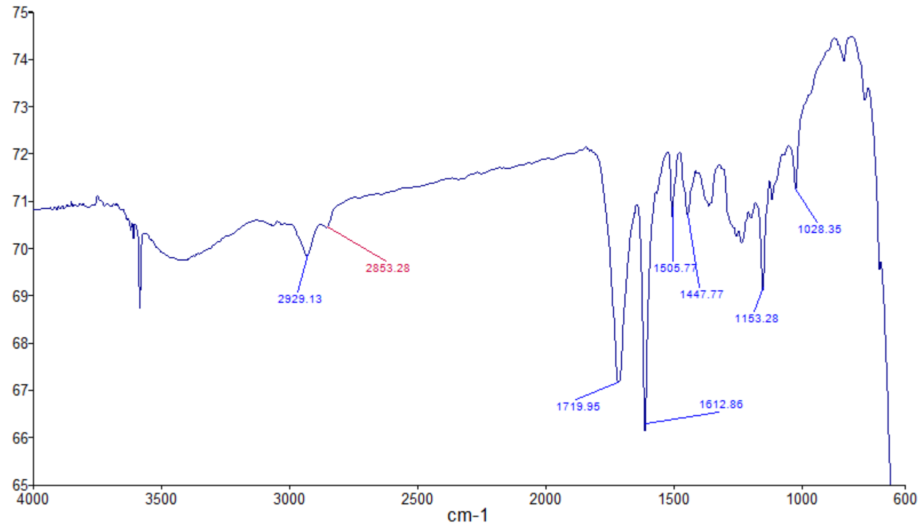

Figure S11 - FTIR (KBr) of co-oligomer **2b**.

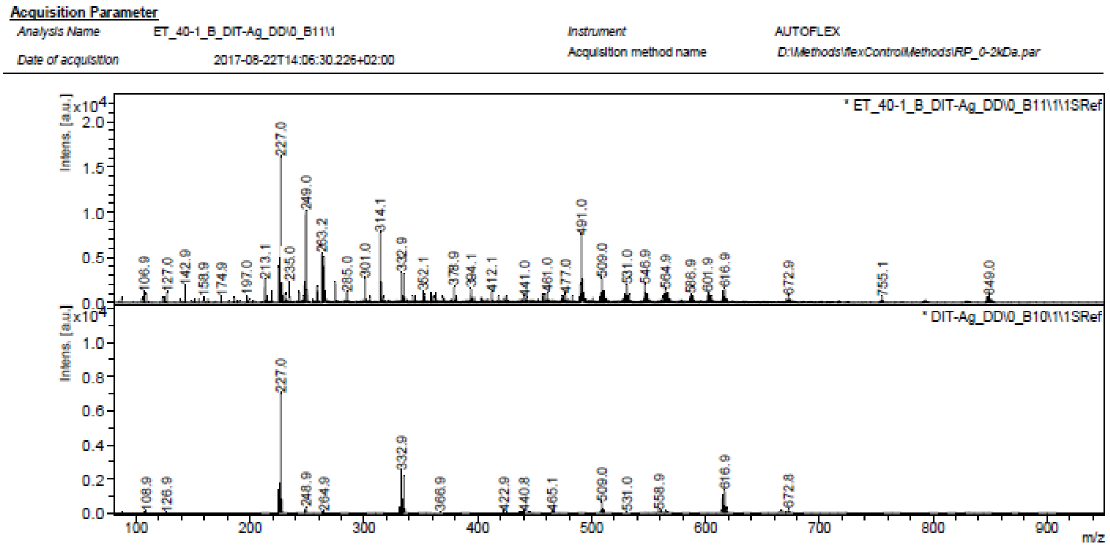

Figure S12 - MALDI-TOF Mass spectrum (matrix: Dithranol + Ag) of co-oligomer **2b**. Possible constitution of ion m/z 849 can be attributed to five molecules of styrene, one of coumarin **2**, a terminal isobutyronitrile and sodium chloride.

Co-oligomer of methyl acrylate with 7-methoxy-3-vinylcoumarin (2a)

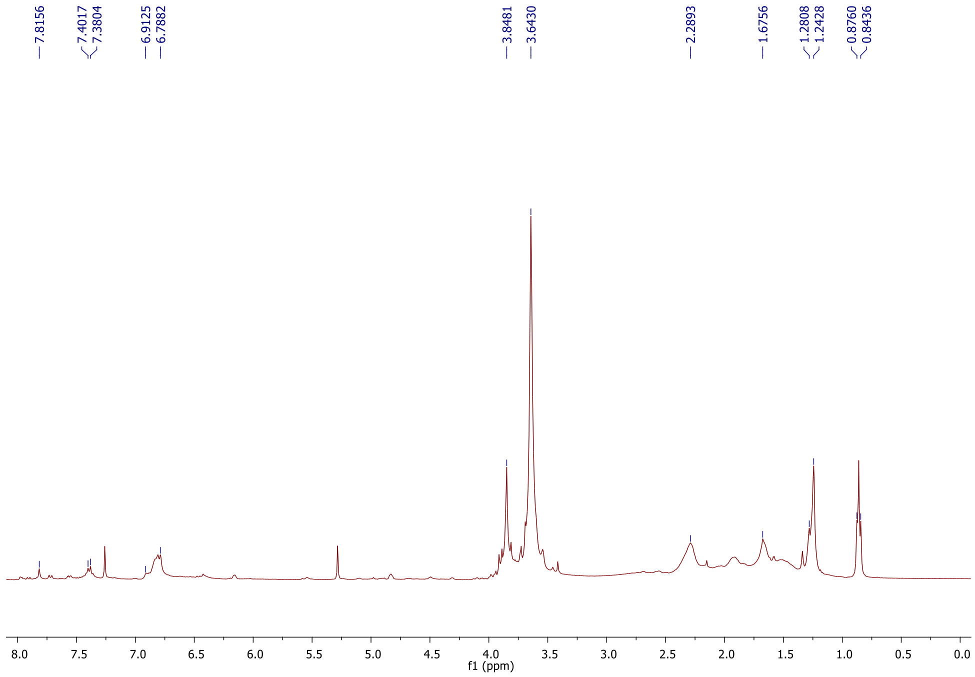

Figure S13 - ^{1}H (400 MHz, $CDCl_3$) of co-oligomer **2a**.

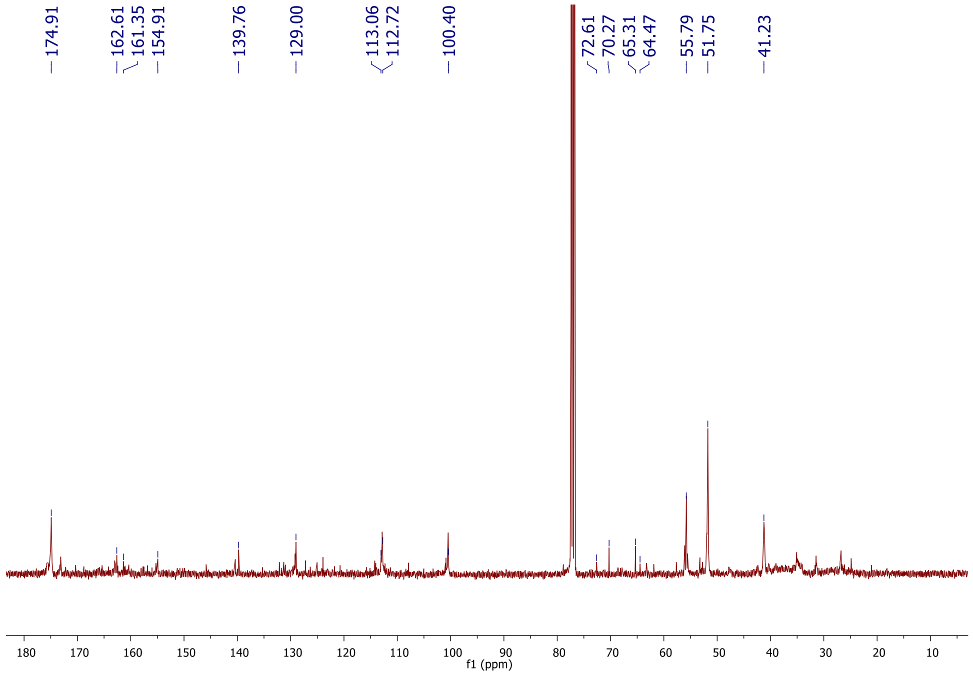

Figure S14 - ^{13}C (100 MHZ, $CDCl_3$) of co-oligomer **2a**.

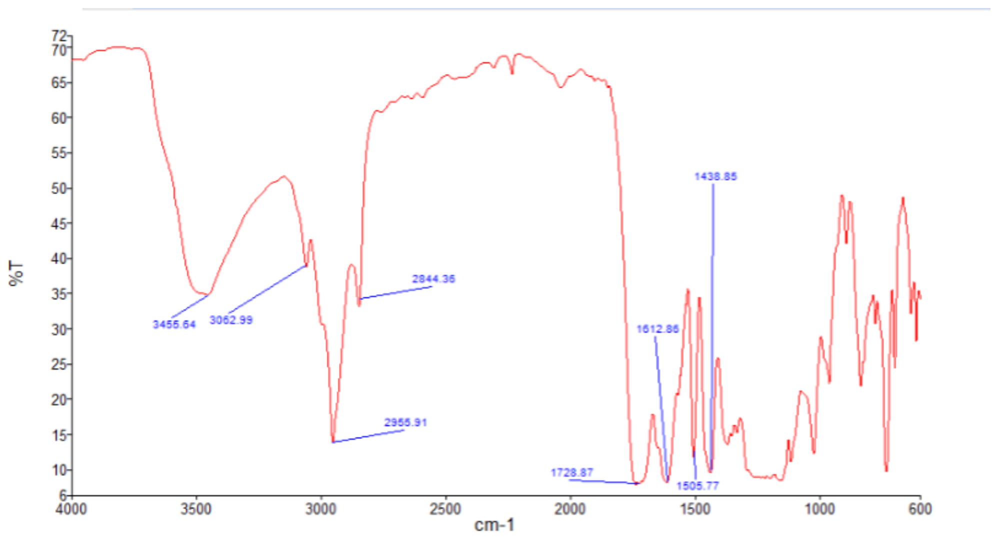

Figure S15 - FTIR (KBr) of co-oligomer **2a.**

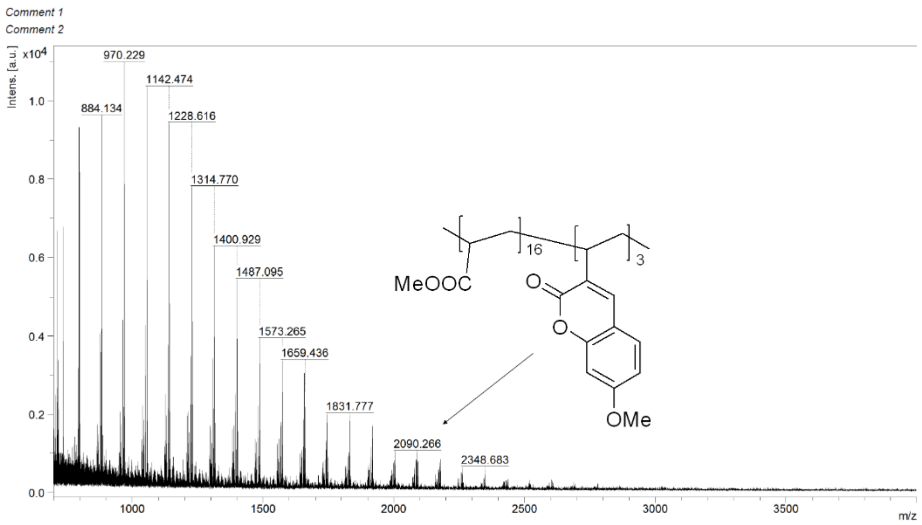

Figure S16 - Mass spectrum MALDI-TOF (DHB matrix) of co-oligomer **2a**. Possible constitution of ion m/z 2090 can be attributed to sixteen molecules of methyl acrylate, three of coumarin **2**, a terminal isobutyronitrile and a potassium ion.

Co-oligomer of methyl acrylate with 7-hydroxy-3-vinylcoumarin (**3a**)

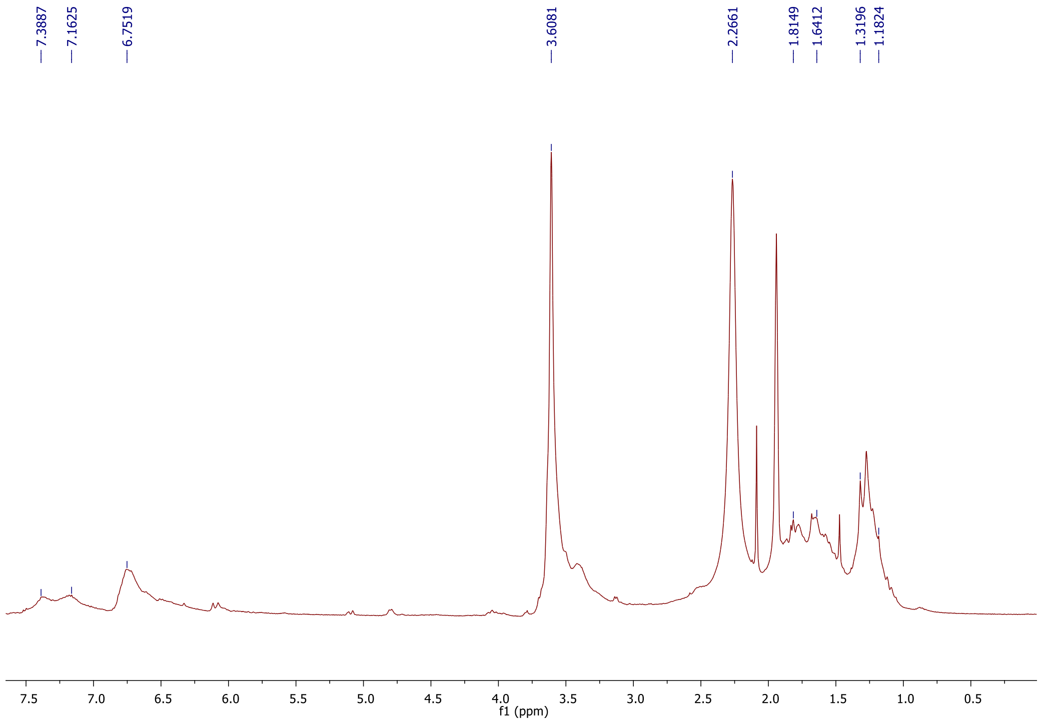

Figure S17 - ^{1}H (400 MHz, CD_3CN) of co-oligomer **3a**.

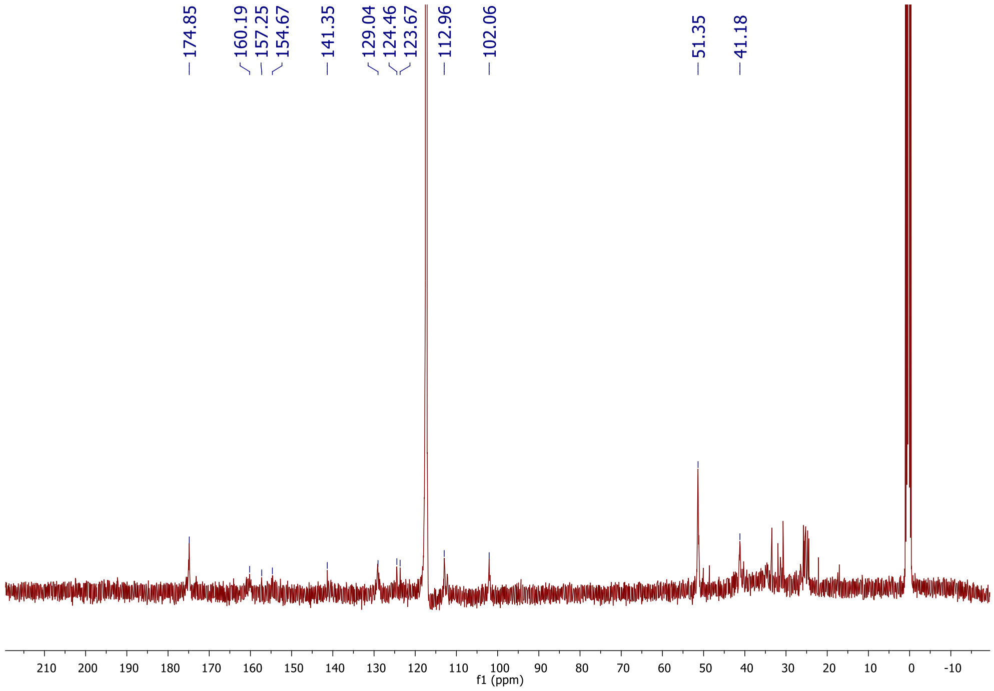

Figure S18 - ^{13}C (100 MHZ, CD_3CN) of co-oligomer **3a**.

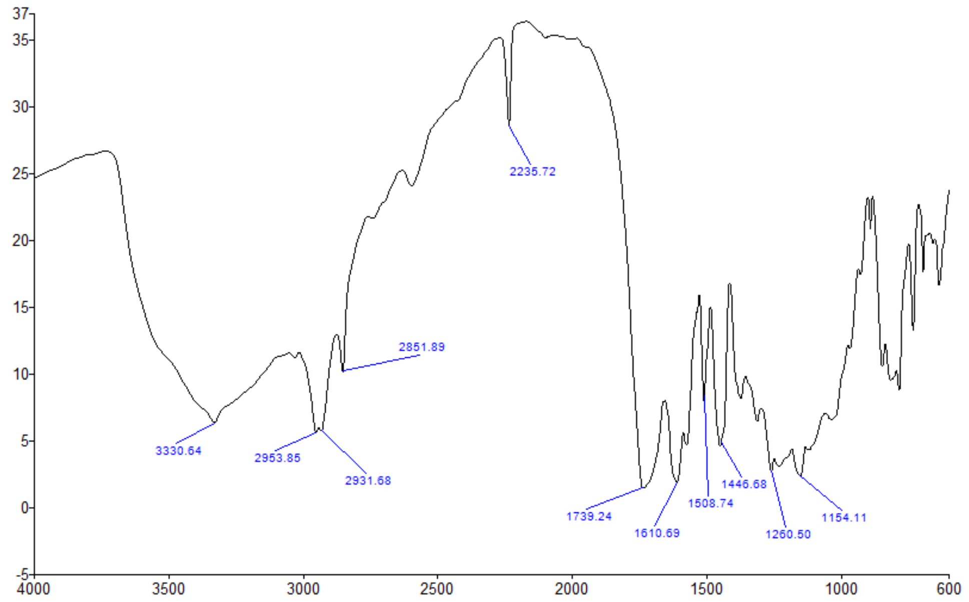

Figure S19 - FTIR (KBr) of co-oligomer **3a**.

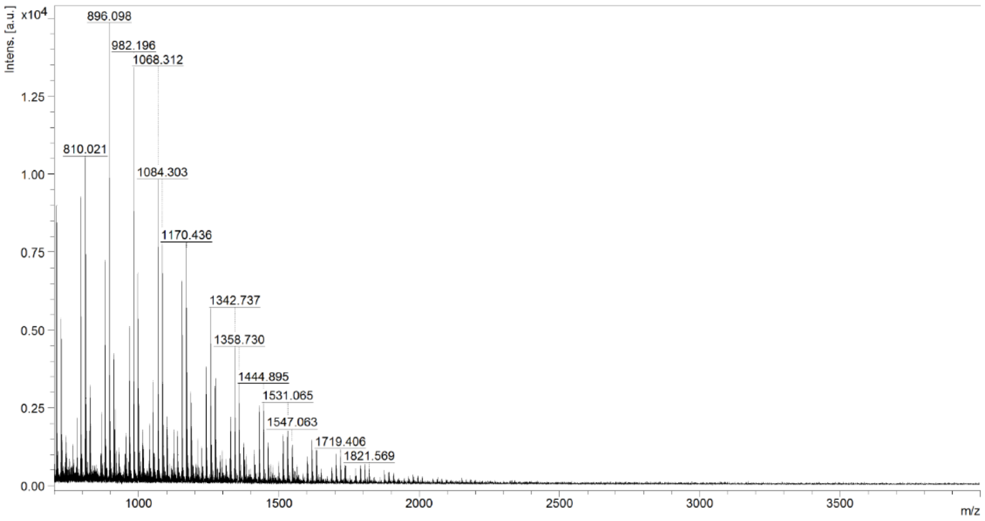

Figure S20 - Mass spectrum MALDI-TOF (DCTB matrix) of co-oligomer **3a**. Possible constitution of ion m/z 1531 can be attributed to ten molecules of methyl acrylate, two of coumarin **3**, a terminal isobutyronitrile and a potassium ion.

Co-oligomer of methyl acrylate with 3-vinylcoumarin (**1a**)

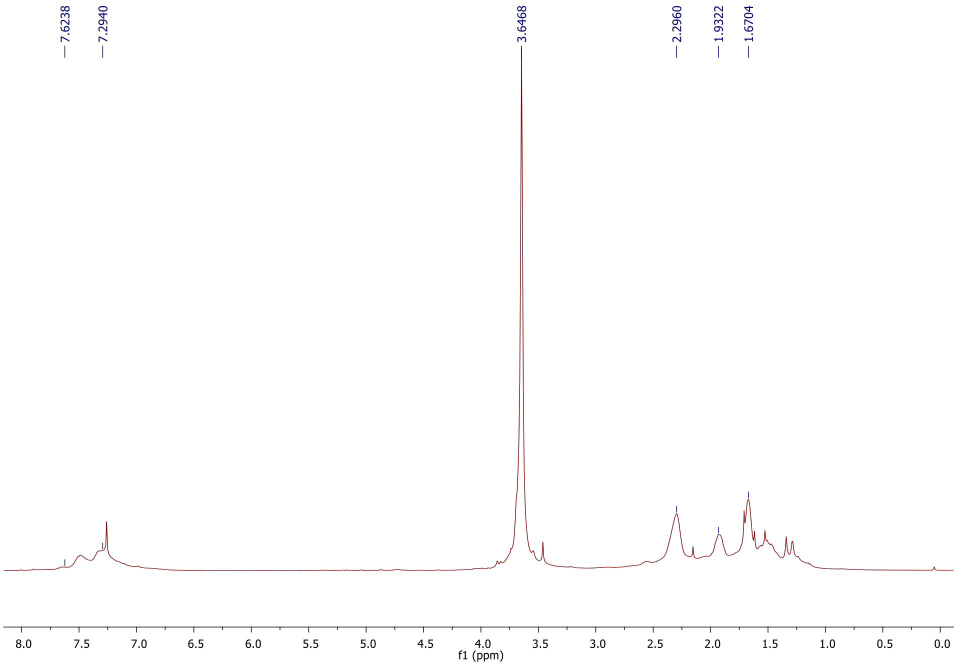

Figure S21 - ^{1}H (400 MHz, $CDCl_3$) of co-oligmer **1a**.

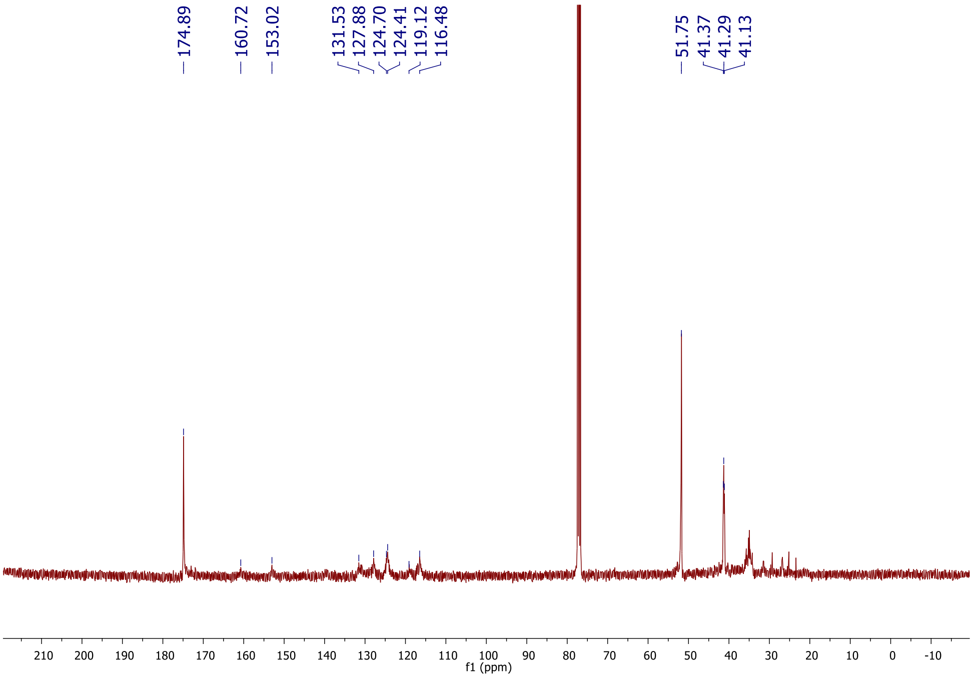

Figure S22 - ^{13}C (100 MHZ, $CDCl_3$) of co-oligmer **1a**.

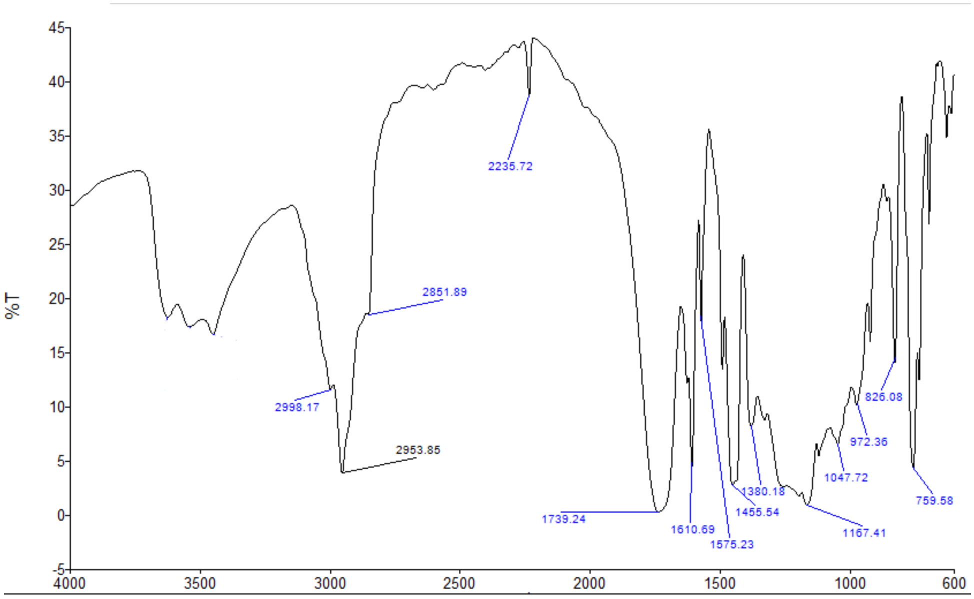

Figure S23 - FTIR (NaCl) of co-oligmer **1a**.

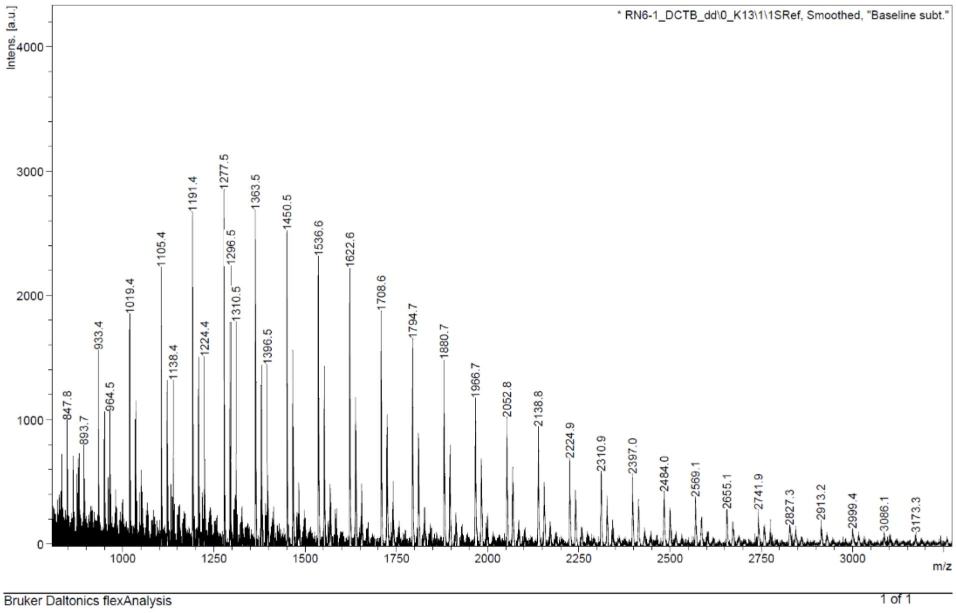

Figure S24 - Mass spectrum MALDI-TOF (DCTB matrix) of co-oligmer **1a**.
